# Optimization of an Experimental Vaccine To Prevent Escherichia coli Urinary Tract Infection

**DOI:** 10.1128/mBio.00555-20

**Published:** 2020-04-28

**Authors:** Valerie S. Forsyth, Stephanie D. Himpsl, Sara N. Smith, Christina A. Sarkissian, Laura A. Mike, Jolie A. Stocki, Anna Sintsova, Christopher J. Alteri, Harry L. T. Mobley

**Affiliations:** a Department of Microbiology and Immunology, University of Michigan Medical School, Ann Arbor, Michigan, USA; b Department of Natural Sciences, University of Michigan—Dearborn, Dearborn, Michigan, USA; Washington University; University of Michigan-Ann Arbor; UTMB; University of Maryland School of Medicine

**Keywords:** CpG, *Escherichia coli*, FyuA, Hma, IreA, IutA, MPLA, vaccine, alum, dmLT, polyIC, urinary tract infection

## Abstract

Urinary tract infections (UTI) affect half of all women at least once during their lifetime. The rise in the numbers of extended-spectrum beta-lactamase-producing strains and the potential for carbapenem resistance within uropathogenic Escherichia coli (UPEC), the most common causative agent of UTI, create an urgent need for vaccine development. Intranasal immunization of mice with UPEC outer membrane iron receptors FyuA, Hma, IreA, and IutA, conjugated to cholera toxin, provides protection in the bladder or kidneys under conditions of challenge with UPEC strain CFT073 or strain 536. On the basis of these data, we sought to optimize the vaccination route (intramuscular, intranasal, or subcutaneous) in combination with adjuvants suitable for human use, including aluminum hydroxide gel (alum), monophosphoryl lipid A (MPLA), unmethylated CpG synthetic oligodeoxynucleotides (CpG), polyinosinic:polycytidylic acid (polyIC), and mutated heat-labile E. coli enterotoxin (dmLT). Mice intranasally vaccinated with dmLT-IutA and dmLT-Hma displayed significant reductions in bladder colonization (86-fold and 32-fold, respectively), with 40% to 42% of mice having no detectable CFU. Intranasal vaccination of mice with CpG-IutA and polyIC-IutA significantly reduced kidney colonization (131-fold) and urine CFU (22-fold), respectively. dmLT generated the most consistently robust antibody response in intranasally immunized mice, while MPLA and alum produced greater concentrations of antigen-specific serum IgG with intramuscular immunization. On the basis of these results, we conclude that intranasal administration of Hma or IutA formulated with dmLT adjuvant provides the greatest protection from UPEC UTI. This report advances our progress toward a vaccine against uncomplicated UTI, which will significantly improve the quality of life for women burdened by recurrent UTI and enable better antibiotic stewardship.

## Abstract (precis)

Urinary tract infections (UTI) are among the most common bacterial infection in humans, affecting half of all women at least once during their lifetimes. The rise in antibiotic resistance and health care costs emphasizes the need to develop a vaccine against the most common UTI pathogen, Escherichia coli. Vaccinating mice intranasally with a detoxified heat-labile enterotoxin and two surface-exposed receptors, Hma or IutA, significantly reduced bacterial burden in the bladder. This work highlights progress in the development of a UTI vaccine formulated with adjuvants suitable for human use and antigens that encode outer membrane iron receptors required for infection in the iron-limited urinary tract.

## INTRODUCTION

Urinary tract infections (UTI), representing the second most common form of human infection after respiratory infections, result in an annual cost of $3.5 billion (1, 2). Uropathogenic Escherichia coli (UPEC) is the most prevalent causative agent of uncomplicated UTI, rates of antibiotic resistance in pathogenic isolates are increasing, and multidrug-resistant strains (E. coli ST131) are emerging (1–3). Despite innate immune defenses in the bladder that include micturition, a mucin layer, constitutively expressed secretory immunoglobulin A, cationic antimicrobial peptides, Tamm-Horsfall protein, lactoferrin, and lipocalin-2 (4), half of all women will experience a UTI in their lifetime, with 1 in 40 women experiencing recurrent infections (5). Patients with acute or recurrent UTI have significantly decreased levels of total secretory IgA in the urine compared to healthy individuals with no history of UTI (6, 7). This indicates the potential for decreased severity and duration of infection if microbe-specific antibody levels can be increased with a vaccine. Because 90% of symptomatic UTI are uncomplicated infections, an ideal vaccine will target factors critical for establishment of bladder colonization (3). Five FDA-approved vaccines provide mucosal protection against other pathogens, including poliovirus, rotavirus, influenza virus, Salmonella enterica serovar Typhi, and Vibrio cholerae (8–11). These efficacious mucosal vaccines that protect against other enteric viruses and bacteria bolster the hypothesis that a vaccine effective against uropathogens is attainable.

During the last 20 years there have been noteworthy advancements toward the development of a UTI vaccine, yet no licensed UTI vaccines are available for use in the United States. Published studies have investigated the efficacy of vaccines containing O antigen (12), fimbrial subunits (13, 14), α-hemolysin (15), siderophores (16), and a variety of outer membrane siderophore receptors in animal models of UTI (17–20). Human clinical trials have been performed on three vaccines, Uro-Vaxom, SolcoUrovac, and ExPEC4V. Uro-Vaxom, comprised of 18 E. coli uropathogen extracts and administered as a daily oral tablet, is approved in Germany and Switzerland for the prevention of recurrent cystitis (21). SolcoUrovac, currently marketed as StroVac, contains heat-killed uropathogenic bacteria, including E. coli, Proteus vulgaris, Klebsiella pneumoniae, Morganella morganii, and Enterococcus faecalis, and is approved for human use in Europe (3, 22). ExPEC4V consists of four conjugated O-antigens, O1A, O2, O6A, and O25B, common to E. coli strains known to cause UTI (23). In a study comparing the efficacies of these three vaccines in adults with recurrent UTI, Uro-Vaxom showed the greatest reduction in the rate of UTI recurrence whereas ExPEC4V did not appear to reduce UTI recurrence (24). Nonetheless, the daily regimen and toxic side effects have limited the widespread use of Uro-Vaxom (25).

Here, we describe our efforts to develop a vaccine against uncomplicated UTI using antigens previously identified and validated as vaccine candidates by intranasal immunization in a murine UTI model using conjugation to the adjuvant cholera toxin (19, 20, 26). We previously employed an extensive multi-omics approach to identify genes and their proteins that (i) are localized to the bacterial cell surface (27); (ii) are expressed during growth in human urine (28), murine infection (29), and human infection (30, 31); (iii) possess immunoreactive properties (32); and (iv) are more prevalent in UPEC isolates than in commensal isolates (33, 34). A total of four β-barrel outer membrane receptors required for iron acquisition, including heme receptor Hma, aerobactin receptor IutA, yersiniabactin receptor FyuA, and putative siderophore receptor IreA, met all of these criteria. Effective iron acquisition from the iron-limited environment of the urinary tract is required for full virulence of UPEC (35–37). In addition to an iron scavenging function, IreA functions as an adhesin that is important for colonization of the bladder (36) and FyuA plays a role in biofilm formation in human urine (38). Intranasal immunization with Hma, IreA, IutA, or FyuA, conjugated to cholera toxin, significantly reduced bacterial burden in the bladder or kidneys or both 48 h following transurethral challenge with UPEC (19, 20). While cholera toxin is an effective immune stimulant in mice, it is not suitable for human use due to development of profuse diarrhea with oral doses as low as 5 μg (39). Because of this drawback, we sought to optimize this UTI vaccine by incorporating adjuvants approved for use in humans or used in vaccine clinical trials.

The precise immune response required for protection against UTI is not well defined. Therefore, we selected a panel of adjuvants known to elicit an array of adaptive immune responses, with the aim being to identify an adjuvant that is well suited for protecting against UTI and safe for use in humans (3, 40). The five adjuvants tested were aluminum hydroxide gel (alum) (41, 42), monophosphoryl lipid A (MPLA) (43–45), unmethylated CpG synthetic oligodeoxynucleotides (CpG) (46–48), polyinosinic:polycytidylic acid (polyIC) (49), and double mutant (R192G/L211A) heat-labile E. coli enterotoxin (dmLT) (50). Alum is licensed for use in 22 vaccines available in the United States and was previously reported to activate dendritic cells via multiple mechanisms, thus promoting antigen uptake and release of interleukin 1β (IL-1β) and IL-18 (41, 51). MPLA is derived from Salmonella enterica serovar Minnesota R595 lipopolysaccharide, activates cellular immunity through the Toll-like receptor 4 (TLR4) signaling pathway, is approved for human use in Europe, and is a component of vaccines for hepatitis B and papillomaviruses (43, 49). CpG activates TLR9 signaling in B cells and dendritic cells, increases mucosal immune responses, and is licensed for use in a hepatitis B vaccine (44, 45, 52). Both dmLT and CpG are presumed to function by activating innate signaling and stimulating mucosal dendritic cells, which activate the adaptive immune response, particularly that of Th17 cells, by dmLT (50, 53–55). polyIC is a synthetically produced double-stranded RNA, analogous to viral RNA, that induces a robust type I interferon response resulting in activation of cellular immunity and is in late-stage clinical development (49).

In an effort to develop a vaccine protective against uncomplicated UTI in humans, we tested five adjuvants (dmLT, CpG, polyIC, MPLA, and alum) with four antigens (Hma, IreA, IutA, and FyuA) for efficacy in mice. Because the immunization route can affect the immune response, we examined three routes of immunization, intranasal (IN), intramuscular (IM), and subcutaneous (SQ), with multiple antigen-adjuvant combinations. Hma and IreA, which were previously demonstrated to provide the most robust reduction of bacterial burden in the kidneys and bladder (19), respectively, were initially examined under all conditions, and then the most promising combinations of route and adjuvant were further evaluated for efficacy with the remaining two antigens FyuA and IutA. Here, we report that intranasal immunization with dmLT-Hma and dmLT-IutA induced antigen-specific antibody production and provided robust protection in immunized mice following transurethral challenge with UPEC.

## RESULTS

### Immunizing via the intranasal route provides the most protection against UTI.

We previously established that intranasal immunization followed by two weekly boosts with an outer membrane iron receptor, Hma, IreA, IutA, or FyuA, conjugated to cholera toxin significantly reduced bacterial burden in the bladder or kidneys or both 48 h following transurethral challenge with UPEC (19, 20). We later determined that conjugation of antigen to adjuvant was not required for protection (unpublished results). On the basis of these data, we began systematic optimization of the vaccine to maximize efficacy using alternative adjuvants admixed with antigen. The optimized vaccine route, adjuvant, and antigen were evaluated based on three criteria: reduction of bacterial CFU in the sample sites, increased number of mice without detectable bacterial counts, and production of antigen-specific antibody. Genes encoding all protein antigens were codon optimized, and proteins were purified from inclusion bodies and certified lipopolysaccharide (LPS) free.

The immunization route can markedly affect the efficacy of vaccines, and some routes are linked to greater patient compliance, especially when immunization requires multiple boosts (56, 57). In an effort to determine the optimal route, we systematically immunized mice intranasally, intramuscularly, or subcutaneously with one of five adjuvants in combination with either Hma or IreA, each of which has been shown to significantly reduce bacterial burden in the kidneys or bladder, respectively (19). Promising combinations of route and adjuvant were further evaluated for efficacy by immunizing with the remaining two antigens FyuA and IutA. Intramuscular and subcutaneous routes were chosen to expand upon early success with intranasal immunization because they are commonly used deliver vaccines in humans. Mice were immunized as previously described (19). In total, 35 immunization trials utilizing 1,060 mice were performed.

**FIG 1:**
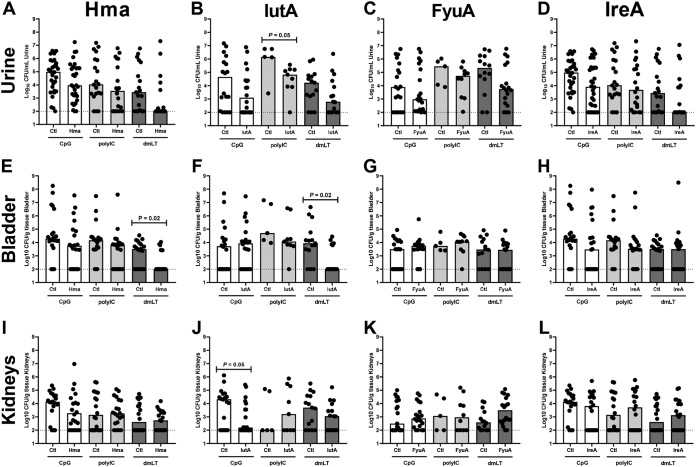
Comparison of adjuvants administered intranasally and formulated with the antigens Hma, IutA, FyuA, and IreA. Seven-to-8-week-old female CBA/J mice were immunized intranasally according to our immunization schedule with adjuvant alone (Ctl) or with adjuvant formulated with 100 μg LPS-free antigen, Hma (A, E, and I), IutA (B, F, and J), FyuA (C, G, and K), or IreA (D, H, and L). Adjuvants tested included unmethylated CpG synthetic oligodeoxynucleotides (CpG), polyinosinic:polycytidylic acid (polyIC), or detoxified E. coli enterotoxin (dmLT). At 1 week after the final boost, mice were challenged with 10^8^ CFU of E. coli strain CFT073 (Hma, IreA, or IutA) or 536 (FyuA) via transurethral inoculation. At 48 h postinoculation, urine was collected, mice were sacrificed, bladder and kidneys were homogenized, and aliquots were plated on LB agar for enumeration of bacterial burden. Bars indicate the median CFU in the urine (A to D), bladder (E to H), and kidneys (I to L). Symbols represent individual mice. *n* = 5 to 30. The dashed line in each panel represents the limit of detection. *P* values were determined using a two-tailed Mann-Whitney test.

We found that intranasal immunization tended to reduce median bacterial burden at least 2-fold in the urine for all antigen-adjuvant combinations tested (Table 1). When immunized intranasally, a 2-fold reduction in median bacterial burden in the bladder occurred for 58% (7/12) of combinations tested and in the kidneys for 33% (4/12) of combinations tested (Table 1). Significant differences are noted in bold in Table 1. Specifically, intranasal immunization with polyIC-IutA (*P = *0.059 urine), dmLT-Hma (*P = *0.024 bladder), dmLT-IutA (*P = *0.018 bladder), and CpG-IutA (*P = *0.046 kidneys) significantly reduced bacterial burden (Fig. 1B, E, F, and J) (Table 1). Three adjuvant-antigen combinations tended to reduce CFU more than 2-fold in all sites of infection following intranasal immunization: dmLT-IutA, CpG-Hma, and CpG-IreA (Table 1). The bimodal distribution of bladder colonization observed with protective combinations (dmLT-Hma, dmLT-IutA) is typical of this model (19, 20).

**TABLE 1:** Median fold change in CFU in the urine, bladder, and kidneys of immunized mice

Route	Adjuvant	Antigen	Urine				Bladder				Kidneys			
			Median Adj only^*a*^	Median Adj + antigen^*b*^	Fold change^*c*^	*P* value^*d*^	Median Adj only	Median Adj + antigen	Fold change	*P* value	Median Adj Only	Median Adj + antigen	Fold change	*P* value
IM^*e*^	Alum	Hma	1,840	105300	0.02	0.0257	100	8v670	0.01	0.2628	10,940	6,860	1.59	0.9861
IM	Alum	IreA	1,840	7,285	0.25	0.0664	100	2,950	0.03	0.9709	10,940	10,045	1.09	0.5761
IM	PolyIC^*f*^	Hma	23,100	734,500	0.03	0.4973	6,735	37,350	0.18	0.0522	9,650	3,975	**2.43**	0.2834
IM	PolyIC	IreA	23,100	106,000	0.22	0.6421	6,735	13,550	0.50	0.5326	9,650	750	**12.87**	0.1808
IM	MPLA^*g*^	Hma	105,350	562,500	0.19	0.4813	100	4,810	0.02	0.3285	3,455	13,620	0.25	0.3888
IM	MPLA	IreA	105,350	17,100	**6.16**	0.2466	100	1,690	0.06	0.7799	3,455	470	**7.35**	0.8994
IM	dmLT^*h*^	Hma	218,500	101,100	**2.16**	0.3440	28,200	92,650	0.30	0.4311	40,400	6,650	**6.08**	0.2400
IM	dmLT	IreA	218,500	36,500	**5.99**	0.1127	28,200	14,500	1.94	0.7275	40,400	11,000	**3.67**	0.7252
IN^*i*^	PolyIC	Hma	10,950	3,500	**3.13**	0.2409	14,600	6,355	**2.30**	0.1009	1,409	1,810	0.78	>0.9999
IN	PolyIC	IreA	10,950	4,845	**2.26**	0.4122	14,600	3,330	**4.38**	0.1457	1,409	5,125	0.27	0.7937
IN	PolyIC	FyuA	284,000	54,100	**5.25**	0.3097	5,500	11,800	0.47	0.5941	1,180	927	1.27	>0.9999
IN	PolyIC	IutA	1440,000	66,700	**21.59**	**0.0599**	50,900	11,800	**4.31**	0.1469	640	1,600	0.40	0.5804
IN	dmLT	Hma	2,790	100	**27.90**	0.0693	3,205	100	**32.05**	**0.0240**	407	542	0.75	0.6033
IN	dmLT	IreA	2,790	105	**26.57**	0.3674	3,205	3,170	1.01	0.9020	407	1,340	0.30	0.7087
IN	dmLT	FyuA	210,450	5,605	**37.55**	0.0825	2,955	2,835	1.04	0.4233	387	3,159	0.12	0.0476
IN	dmLT	IutA	16,400	619	**26.49**	0.1866	8,610	100	**86.10**	**0.0181**	4,950	1,130	**4.38**	0.2536
IN	CpG^*j*^	Hma	97,050	9,285	**10.45**	0.4332	19,350	5,755	**3.36**	0.1321	13,500	1,885	**7.16**	0.1419
IN	CpG	IreA	97,050	8,215	**11.81**	0.4944	19,350	3,035	**6.38**	0.0608	13,500	6,490	**2.08**	0.2991
IN	CpG	FyuA	7,910	961	**8.23**	0.6403	3,130	5,625	0.56	0.8550	312	773	0.40	0.8330
IN	CpG	IutA	44,530	1,240	**35.91**	0.1959	5,255	8,570	0.61	0.7791	21,100	161	**131.06**	**0.0462**
SQ^*k*^	Alum	Hma	2,218	100	**22.18**	0.1246	3,240	8,205	0.39	0.7940	198	100	1.98	0.2391
SQ	Alum	IreA	2,218	3,165	0.70	0.7828	3,240	4,655	0.70	0.9271	198	2,315	0.09	0.3376
SQ	PolyIC	Hma	100	1,705	0.06	0.7168	1,505	1,4650	0.10	0.0052	100	100	1.00	0.4241
SQ	PolyIC	IreA	100	1,710	0.06	0.5473	1,505	4,410	0.34	0.2559	100	9,615	0.01	0.0274
SQ	dmLT	Hma	119,000	8,955	**13.29**	0.3154	2,750	2,555	1.08	0.9478	4,390	12,440	0.35	0.4422
SQ	dmLT	IreA	119,000	213,500	0.56	0.9048	2,750	11,360	0.24	0.7122	4,390	4,990	0.88	0.9675
SQ	MPLA	Hma	31,900	232,600	0.14	0.7988	5,240	13,800	0.38	0.5935	41,800	5,615	**7.44**	0.2818
SQ	MPLA	IreA	31,900	202,000	0.16	0.4002	5,240	14,700	0.36	0.0810	41,800	54,650	0.76	0.2309

a Median CFU when immunized with the adjuvant alone.

b Median CFU when immunized with the adjuvant formulated with antigen.

c Fold change in median CFU when immunized with the adjuvant alone compared to mice immunized with the adjuvant formulated with antigen. Fold changes greater than 2 are shown in bold.

d *P* value as determined by two-tailed Mann-Whitney test. Significant differences are shown in bold.

e Intramuscular.

f Polyinosinic:polycytidylic acid.

g Monophosphoryl lipid A.

h Detoxified E. coli enterotoxin.

i Intranasal.

j Unmethylated CpG synthetic oligodeoxynucleotides.

k Subcutaneous.

Immunization via the intramuscular and subcutaneous routes showed limited protection. Intramuscular immunization utilizing Hma as antigen tended to reduce median bacterial burden at least 2-fold in 3 of 12 trials (Table 1). Immunization with IreA intramuscularly tended to reduce bacterial burden at least 2-fold in 5 out of 12 trials. The use of the subcutaneous route in combination with Hma tended to reduce median CFU levels at least 2-fold in the urine when formulated with alum or dmLT and in the kidneys when formulated with MPLA (Table 1). No reduction in bacterial burden was observed for any of the sites of infection when any of the IreA vaccine formulations were administered subcutaneously (Table 1).

Bacterial inocula for the 35 infection trials were confirmed to be at the desired dose with median doses of 3.05 ± 0.11 × 10^9^ CFU/ml of strain CFT073 and 2.70 ± 0.20 × 10^9^ CFU/ml of strain 536 (see Fig. S1 in the supplemental material). Strain CFT073 lacks a functional FyuA; therefore, vaccine combinations containing this antigen utilized E. coli strain 536 for challenge. These data verify that variances in colonization levels between the experimental trials were not due to differences in the bacterial doses used to transurethrally inoculate mice (Fig. S1). When polyIC or CpG was administered intranasally in the absence of antigen, the median colonization level of CFT073 was not significantly different from that seen with unimmunized mice infected with the same dose of CFT073 (Fig. S2), indicating that polyIC or CpG alone do not alter colonization of CFT073. However, intranasal administration of dmLT as an adjuvant in the absence of antigen significantly reduced the bacterial burden in the kidneys (*P = *0.02) (Fig. S2), consistent with previous findings showing that dmLT alone reduced colonization of multiple pathogens, including Haemophilus influenzae, Campylobacter jejuni, and Shigella flexneri (50).

10.1128/mBio.00555-20.1 FIG S1 Inocula are consistent across experimental trials and strains. Data represent inoculating doses of UPEC (indicated as CFU counts per milliliter) administered transurethrally to female CBA/J mice 1 week following final boost. Mice were inoculated with strain CFT073 following immunization with Hma, IreA, or IutA or with strain 536 following immunization with FyuA. The intended dose was 2 × 10^9^ CFU/ml. Whiskers indicate maximum and minimum, the box indicates 25th and 75th percentiles, the bar indicates the median. *n* = 6 to 26. No statistical difference was found via two-tailed Mann-Whitney test. Download FIG S1, TIF file, 0.1 MB. Copyright © 2020 Forsyth et al. 2020 Forsyth et al. This content is distributed under the terms of the Creative Commons Attribution 4.0 International license.

10.1128/mBio.00555-20.2 FIG S2 Immunizing with CpG or polyIC in the absence of antigen does not affect colonization of the urinary tract by UPEC. Seven-to-eight-week-old female CBA/J mice were immunized intranasally according to our immunization schedule with the following adjuvants: unmethylated CpG synthetic oligodeoxynucleotides (CpG), detoxified E. coli enterotoxin (dmLT), or polyinosinic:polycytidylic acid (polyIC). One week after the final boost, mice were transurethrally challenged with 10^8^ CFU of CFT073. At 48 h postinoculation, urine was collected, mice were sacrificed, bladder and kidneys were homogenized, and aliquots were plated on LB agar for enumeration of bacterial burden. Bars indicate the median CFU in the urine (A), bladder (B), and kidneys (C). Symbols represent individual mice. *n* = 5 to 40. The limit of detection was 100 CFU/ml urine or 100 CFU/g tissue. The dotted line represents colonization level observed in unimmunized mice challenged with CFT073. *P* values shown where addition of adjuvant was protective as determined using a two-tailed Mann-Whitney test. Download FIG S2, TIF file, 0.2 MB. Copyright © 2020 Forsyth et al. 2020 Forsyth et al. This content is distributed under the terms of the Creative Commons Attribution 4.0 International license.

Together, these results indicate that intranasal immunization tended to improve the protective response in urine, bladder, and kidneys at least 2-fold following bacterial challenge for 64% (23/36) of the adjuvant-antigen combinations, with 4 combinations showing statistically significant results, in comparison to intramuscular immunization for 33% (8/24, *P = *0.03) or subcutaneous immunization for 13% (3/24, *P < *0.005) (Table 1). No statistically significant CFU reductions were observed in any combination administered by the intramuscular or subcutaneous route. Therefore, intranasal immunization is the optimal route for protection against transurethral challenge with E. coli.

### dmLT is the adjuvant that provides the most effective protection against colonization by E. coli.

Having determined that intranasally administering different vaccine formulations provided the most consistent protection against UTI, we next set out to optimize the adjuvant. Previous studies have shown that immunizing with cholera toxin conjugated to antigens provides a robust immune response and reduces bacterial burden in the bladder and kidneys of CBA/J mice (19, 20); however, it is not suitable for human use (39). In an effort to develop a vaccine to prevent UTI, we tested our antigen candidates admixed with alternative adjuvants approved for human immunization, i.e., dmLT, CpG, polyIC, MPLA, and alum, for efficacy in mice. Intranasal immunization with dmLT, an adjuvant very similar in structure to cholera toxin (both are A_1_B_5_ toxins), significantly reduced the median level of bladder colonization (*P = *0.02) when admixed with the antigen Hma compared to dmLT alone (Fig. 1E). In addition, this vaccine combination significantly increased the number of mice without detectable bacteria in the bladder (35% dmLT only, 68% dmLT-Hma, *P = *0.056) and urine (26% dmLT only, 61% dmLT-Hma, *P = *0.05), indicating that more mice cleared the infection (below the limit of detection) 48 h postinoculation (see Table S1 in the supplemental material). Similarly to Hma, immunizing with dmLT-IutA significantly reduced the median CFU count per gram bladder (*P = *0.02) (Fig. 1F) and significantly increased the number of protected mice (20% dmLT only, 67% dmLT-IutA, *P = *0.03) (Table S1). In addition, dmLT administered with FyuA and IutA tended to reduce urine bacterial load 2-fold (Table 1).

10.1128/mBio.00555-20.5 TABLE S1 Percentages of immunized mice without detectable CFU following transurethral challenge. Download Table S1, DOCX file, 0.1 MB. Copyright © 2020 Forsyth et al. 2020 Forsyth et al. This content is distributed under the terms of the Creative Commons Attribution 4.0 International license.

Other adjuvant-antigen combinations that resulted in significant reductions in bacterial burden when administered intranasally included polyIC-IutA (*P = *0.05 urine) (Fig. 1B) and CpG-IutA (*P = *0.05 kidneys) (Fig. 1J). The primary target of this vaccine is patients with uncomplicated UTI; therefore, reducing bladder colonization and inflammation (cystitis) is the desired outcome. Since dmLT-IutA and dmLT-Hma both reduced bacterial burdens in the bladder, dmLT was selected for future studies.

### IutA is the optimal antigen when coadministered with dmLT via the intranasal route.

Previous studies with cholera toxin conjugated to antigens found that IreA and IutA provided significant protection from bacterial challenge in the bladder and that Hma, FyuA, and IutA provided protection in the kidneys (19, 20). When administered via the intranasal route and formulated with dmLT, all four antigens individually trended toward reducing bacterial burdens in the urine (Fig. 1A to D). In the bladder, CFU counts per gram tissue were significantly reduced compared to administration of adjuvant alone when Hma (32-fold reduction, *P = *0.024) and IutA (86-fold reduction, *P = *0.018) were coadministered with dmLT (Fig. 1E and F). FyuA and IreA did not provide any protection compared to the adjuvant-alone cohort (Table 1) (Fig. 1G and H). IutA was the only antigen that reduced colonization more than 2-fold in the kidneys, although this reduction was not statistically significant (Table 1). According to these results, IutA was an effective antigen for reduction of bacterial burden when administered via the intranasal route in combination with dmLT in all sites of infection. In comparison, Hma was an effective antigen in two sites of infection.

### Antigen-specific antibody response to immunization.

**FIG 2:**
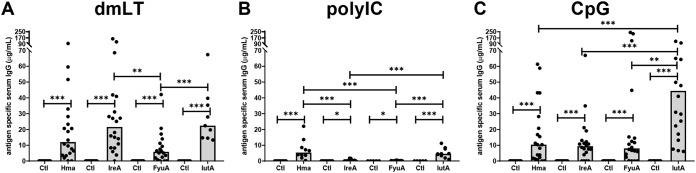
Intranasal vaccination with outer membrane iron receptors generated a robust antigen-specific serum IgG response. Antigen-specific IgG concentrations were quantified by indirect ELISA in serum collected from female CBA/J mice 1 week after final boost. Mice were intranasally immunized with adjuvant alone (Ctl) or adjuvant formulated with 100 μg of purified, LPS-free antigen (Hma, IreA, FyuA, or IutA). Adjuvants utilized were (A) detoxified E. coli enterotoxin, dmLT, (B) polyinosinic:polycytidylic acid, polyIC, and (C) unmethylated CpG synthetic oligodeoxynucleotides, CpG. Each bar represents the median, and each symbol represents an individual mouse. *n* = 5 to 20. *P* values were determined using a two-tailed Mann-Whitney test. ***, *P* ≤ 0.001; **, *P* ≤ 0.01; *, *P* ≤ 0.05.

**FIG 3:**
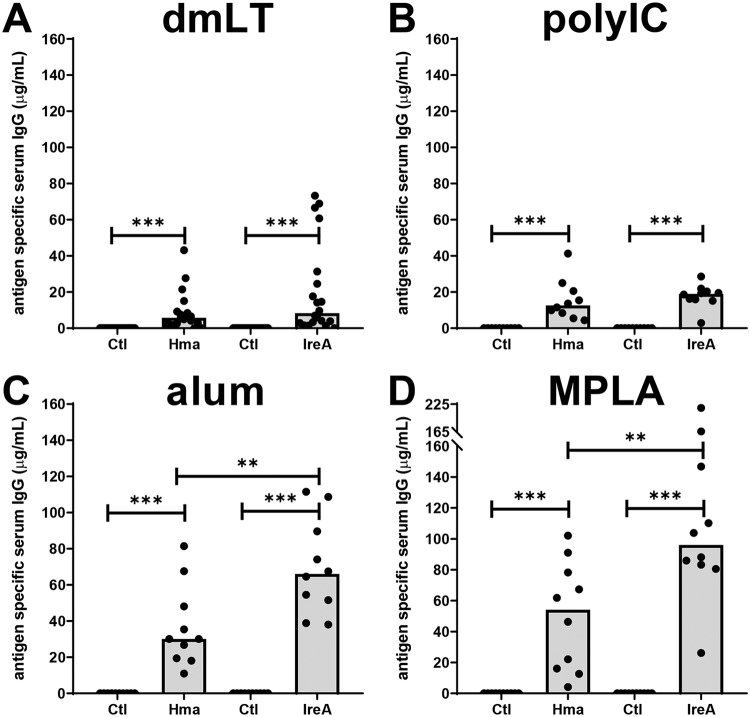
Intramuscular vaccination with outer membrane iron receptors generated a robust antigen-specific serum IgG response when alum or MPLA was used as an adjuvant. Antigen-specific IgG concentrations were quantified by indirect ELISA in serum collected from female CBA/J mice 1 week after final boost. Mice were intramuscularly immunized with adjuvant alone (Ctl) or with adjuvant formulated with 100 μg of purified, LPS-free antigen (Hma, IreA, FyuA, or IutA). Adjuvants utilized were (A) detoxified E. coli enterotoxin, dmLT, (B) polyinosinic:polycytidylic acid, polyIC, (C) alum, and (D) monophosphoryl lipid A, MPLA. Note the change in scale in panel D. Each bar represents the median, and each symbol represents an individual mouse. *n* = 10 to 20. *P* values were determined using a two-tailed Mann-Whitney test. ***, *P* ≤ 0.001; **, *P* ≤ 0.01.

In addition to evaluation of vaccine efficacy by observation of changes in the bacterial burden after challenge, the humoral response was evaluated using an indirect enzyme-linked immunosorbent assay (ELISA). The amount of antigen-specific IgG in serum collected 1 week after the second boost was quantified. In all trials, independent of route, adjuvant, or antigen, mice immunized with adjuvant alone had no measurable amounts of antigen-specific antibody (Fig. 2 and 3; see also Fig. S3). However, mice immunized with adjuvant admixed with antigen showed a statistically significant increase in the concentration of antigen-specific IgG in the serum compared to controls immunized with adjuvant alone (Fig. 2 and 3; see also Fig. S3). This indicated that addition of adjuvant alone does not produce an antigen-specific immune response and that outer membrane iron receptor preparations containing no LPS contamination are antigenic.

10.1128/mBio.00555-20.3 FIG S3 Subcutaneous vaccination with outer membrane iron receptors generates an antigen-specific serum IgG response. Antigen-specific IgG concentrations quantified by indirect ELISA in pooled serum collected from female CBA/J mice 1 week after final boost. Mice were subcutaneously immunized with adjuvant alone (Ctl) or with adjuvant formulated with 100 μg of purified, LPS-free antigen (Hma, IreA, FyuA, and IutA). The adjuvants utilized were as follows: detoxified E. coli enterotoxin, (dmLT), polyinosinic:polycytidylic acid (polyIC), alum, and monophosphoryl lipid A (MPLA). Each bar represents the mean of results from four technical replicates of serum pooled from 10 mice from either one or two experimental trials, and error bars indicate standard deviations. *P* values were determined using a two-tailed Mann-Whitney test. **, *P* ≤ 0.01; *, *P* ≤ 0.05. Download FIG S3, TIF file, 0.1 MB. Copyright © 2020 Forsyth et al. 2020 Forsyth et al. This content is distributed under the terms of the Creative Commons Attribution 4.0 International license.

The immune responses generated between routes of immunization and adjuvants varied. Intranasal immunization with dmLT generated the most consistent and robust serum antibody response across all antigens, with median concentrations of antigen-specific IgG of 12.0 μg/ml for Hma, 21.6 μg/ml for IreA, 5.8 μg/ml for FyuA, and 22.4 μg/ml for IutA (Fig. 2A). In comparison to vaccine formulations that significantly reduced bacterial burden in the bladder of challenged mice, there was no correlation between antigen-specific antibody production and protection for individual mice, indicating that the mechanism of protection may include cell-mediated immunity (Fig. S4). In comparison to immunizations with dmLT, polyIC in combination with all antigens produced a weak antigen-specific antibody response with an average concentration of 3.2 μg/ml antigen-specific IgG (Fig. 2B). Comparing all of the intranasal immunizations, mice immunized intranasally with CpG-IutA produced the greatest median concentration of antigen-specific antibody of all intranasal immunizations (44.9 μg/ml), although the responses were highly variable between individual mice (Fig. 2C) and did not correlate with a reduction in bladder bacterial burden (Fig. S3D).

10.1128/mBio.00555-20.4 FIG S4 Antigen-specific serum antibodies do not correlate with protection against UTI. The data representing CFU per milliliter urine or per gram tissue (*y* axis) of all vaccination trials that significantly reduced bacterial burden (see Fig. 1 and 3) were correlated with antigen-specific serum IgG concentrations measured by ELISA (*x* axis). Seven-to-eight-week-old female CBA/J mice were immunized intranasally according to our immunization schedule with detoxified E. coli enterotoxin (dmLT) formulated with Hma (A), dmLT-IutA (B), polyinosinic:polycytidylic acid formulated with IutA (C), or unmethylated CpG synthetic oligodeoxynucleotides formulated with IutA (D). Each symbol represents a single mouse. Open symbols represent mice immunized with adjuvant alone; closed symbols represent mice immunized with adjuvant formulated with antigen. *n* = 5 to 17. No significant correlations were found using Pearson’s correlation coefficient. Download FIG S4, TIF file, 0.2 MB. Copyright © 2020 Forsyth et al. 2020 Forsyth et al. This content is distributed under the terms of the Creative Commons Attribution 4.0 International license.

Although intramuscular and subcutaneous immunizations were less effective at reducing the bacterial burden in the urine, bladder, and kidneys of challenged mice, these routes were able to generate an antigen-specific IgG response in the serum (Fig. 3; see also Fig. S3D). Intramuscular immunizations with MPLA produced the highest median concentration of antigen-specific antibody for all vaccine trials (median for Hma, 54.1 μg/ml; median for IreA, 96.0 μg/ml) (Fig. 3D), with vaccine combinations that used alum as an adjuvant producing similar responses (Fig. 3C). Immunizations performed with dmLT as an adjuvant resulted in a very weak IgG response when administered intramuscularly (median for Hma, 5.7 μg/ml; median for IreA, 8.3 μg/ml) (Fig. 3A). Subcutaneous immunizations produced a similar antigen-specific IgG response independently of adjuvant, with mean concentrations in pooled mouse sera ranging from 37.8 μg/ml to 97.3 μg/ml (Fig. S3). Most notably, for both the intramuscular and subcutaneous routes, immunization of mice with IreA increased the concentration of antigen-specific antibody above that seen with similar vaccine formulations where Hma was used as the antigen (Fig. 3; see also Fig. S3D), indicating that IreA may be more antigenic despite having a highly similar tertiary structure.

## DISCUSSION

Currently, there is no vaccine licensed in the United States to prevent UTI by E. coli. In this current era of increasing antibiotic resistance, developing preventive measures against urinary tract pathogens, especially UPEC, is critical. With that goal in mind, here we systematically tested four antigens, previously validated for their protective efficacy against colonization of the bladder and kidneys during experimental murine UTI (19, 20), in combination with each of five adjuvants, compatible with the eventual clinical trial design, delivered by each of three routes. Intranasal immunization is protective in mice, while intramuscular or subcutaneous routes are more commonly used in humans. Intranasal administration of dmLT formulated with Hma or IutA significantly reduced bladder CFU levels and significantly increased the number of mice without detectable CFU. Immunization with dmLT, which, like cholera toxin, is an A_1_B_5_ toxin, consistently generated a robust IgG antibody response against the administered antigen. Optimization of the route, adjuvant, and antigen is an important step in development of a UTI vaccine for human clinical trials.

Induction of mucosal immune responses is most efficiently stimulated by delivery of a vaccine at a mucosal surface (58) and may be due to activity of the common mucosal immune system that links tissues of the lung, gastrointestinal tract, urogenital tract, and nasopharynx (59). In mice, monkeys, and humans, vaccines administered intranasally generate mucosal immune responses in the female genital tract (60–63). This is attributed to the expression of redundant B and T cell homing receptors (CCR10, CCL28, and α_4_β_7_ integrin) throughout mucosal surfaces, allowing circulating activated B and T cells to be attracted to multiple mucosal sites (64). Here, we have shown that intranasal immunization is more effective at reducing bacterial CFU in the bladder of mice. Intramuscular and subcutaneous routes of vaccination may be less effective because homing receptors are not expressed on B cells that are activated in peripheral lymph nodes (65). However, it has been shown that subcutaneous immunization performed with 2 weeks between boosts can significantly reduce kidney colonization and generate serum IgG in BALB/c mice when formulated with MPLA-FimH/MrpH (66), alum-FyuA (18), and adjuvant-IroN (17).

Administered intranasally, the adjuvant dmLT significantly reduced colonization of UPEC in the bladder during UTI. Other adjuvants were unable to produce equivalent results, independently of the administration route. The success of dmLT recapitulates previous findings in experiments testing antigenicity of outer membrane iron receptors that utilized cholera toxin conjugated to antigens (19, 20). This is likely related to the similarity between the two toxins. Both are A_1_B_5_ toxins whose B subunits bind to ganglioside GM1 and facilitate endocytosis of the A subunit into the cytosol (67–69). In addition, we found that administration of dmLT alone reduced colonization in the kidneys, consistent with other vaccines where dmLT was used as an adjuvant (50). The mechanism of immune modulation for dmLT has been demonstrated to consist of a strong IL-17 response and activation of Th17 cells (50, 70), which may contribute to its success given the critical role of Th17 cells and IL-17 signaling during the host response to bladder infection (4, 19, 71).

The four antigens used to determine the optimal route and adjuvant for a urinary tract vaccine have similar cellular functions. These proteins display similar β-barrel structures, are located in the Gram-negative bacterial outer membrane, and mediate the uptake of siderophores (FyuA, IutA, and IreA) or heme molecules (Hma) from the extracellular environment. However, despite these similarities, there are marked differences in host responses when they are individually formulated with a single adjuvant. IutA was the only antigen to significantly reduce bacterial colonization in all sites of infection assessed. In addition, intranasal immunization with IutA produced the most robust serum IgG response independently of adjuvant. This could have been due to the high percentage of its amino acid sequence likely to represent major histocompatibility complex (MHC) class II epitopes predicted by BepiPred-2.0 compared to Hma, IreA, and FyuA. Furthermore, IutA was previously shown to be highly expressed in the mouse model of ascending UTI, as assessed by RNA microarray, and in women presenting with symptoms of cystitis (29, 30). Indeed, transcriptome sequencing (RNA-seq) data from 14 different strains, stabilized immediately following urine collection, clearly demonstrated that *iutA*, *hma*, *fyuA*, and *ireA* are highly expressed *in vivo* in women with acute cystitis (72). On the basis of its efficacy and antigenicity, IutA should be considered for inclusion in future UTI vaccines, particularly those aimed at increasing the breadth of protection against UPEC strains and overcoming the functional redundancy of iron receptors by incorporation of multiple antigens.

Traditional vaccine development has been focused on production of antibodies and immunological memory. Our intranasally administered vaccine using the adjuvant dmLT significantly reduces E. coli colonization of the murine bladder when formulated with Hma or IutA; however, serum antigen-specific antibody levels did not correlate with CFU in the current study using purified LPS-free proteins. These results might suggest that administration of adjuvants via our tested routes did not elicit an effective antibody response and that cellular immunity may be involved in protection. To that point, we previously demonstrated that intranasal immunization with bacterial siderophores (yersiniabactin and aerobactin), which bind to our iron-receptor antigens, provided protection by an unknown non-antibody-mediated mechanism (16). In addition, experiments vaccinating mice with cholera toxin conjugated to outer membrane iron receptors generated antigen-specific antibody universally, independently of any observed reduction in CFU during murine ascending UTI (19). Together, these data suggest that a strong IgG antibody response is not indicative of protection against infection. Further work is required to identify the immune factors contributing to protection, which will accelerate the development of an effective human UTI vaccine.

## MATERIALS AND METHODS

### Ethics statement (animal protocols).

All animal protocols were approved by the Institutional Animal Care and Use Committee (IACUC) at the University of Michigan Medical School (PRO00009173) and in accordance with the Office of Laboratory Animal Welfare (OLAW), the United States Department of Agriculture (USDA), and the guidelines specified by the Association for Assessment and Accreditation of Laboratory Animal Care, International (AAALAC, Intl.). Mice were anesthetized with a weight-appropriate dose (0.1 ml for a mouse weighing 20 g) of ketamine/xylazine (80 to 120 mg ketamine/kg of body weight and 5 to 10 mg xylazine/kg) by intraperitoneal injection. Mice were euthanized by inhalant anesthetic overdose followed by vital organ removal.

### Bacterial strains and culture conditions.

E. coli CFT073 was isolated from the blood and urine of a hospitalized patient with acute pyelonephritis and urosepsis (73), and E. coli 536 was isolated from a patient with pyelonephritis (74). Transurethral infections with strain CFT073 or strain 536 were performed in mice immunized with vaccine formulations containing Hma, IreA, IutA, or FyuA. E. coli CFT073 does not encode a functional FyuA protein; thus, strain 536 was used to challenge mice that were immunized with FyuA. Strains were cultured in lysogeny broth (LB) (10 g/liter tryptone, 5 g/liter yeast extract, 0.5 g/liter NaCl) at 37°C with aeration until saturation or on LB agar at 37°C.

### Antigen purification and concentration.

Commercially produced purified antigens were supplied by GenScript as follows. DNA sequences of *hma*, *ireA*, and *iutA* from E. coli CFT073 and *fyuA* from E. coli 536 were codon optimized and individually synthesized in-frame with a 6× histidine affinity tag and subcloned into E. coli expression vector pET-30a. Recombinant plasmids were transformed into E. coli BL21 star (DE3), cultured with shaking at 37°C in TB medium containing kanamycin, induced with IPTG (isopropyl-β-d-thiogalactopyranoside), and harvested by centrifugation at 8,000 rpm. Cell pellets were lysed by sonication. Following centrifugation at 13,000 rpm, the precipitate was dissolved using 8 M urea. Target protein was filter sterilized using a 0.22-μm-pore-size membrane and quantified by the Bradford protein assay, and protein purity was determined by SDS-PAGE and Western blotting. Purified protein was obtained from inclusion bodies, with purity ranging from 85% to 90%. Proteins were certified to be endotoxin free with levels of <100 endotoxin units (EU)/mg. Prior to immunization and subsequent boosts, protein was concentrated with 10,000 nominal molecular weight limit (NMWL) centrifugal filter units (EMD Millipore).

### Vaccine formulation and administration.

Formulation of vaccines by admixing antigen and adjuvant was performed on the day of primary immunization and on the day of each subsequent boost. For each trial, 7-to-8-week-old CBA/J mice were given a primary dose of adjuvant alone or adjuvant combined with 100 μg purified, LPS-free antigen via the specified route, intramuscular (IM), subcutaneous (SQ), or intranasal (IN), on day 0. On days 7 and 14, mice were boosted with adjuvant alone or with adjuvant combined with 25 μg antigen by the same route. On day 21, blood was collected retro-orbitally via capillary tube, and mice were transurethrally inoculated with a UPEC strain expressing the antigen of interest. At 48 h postinoculation, urine was collected via abdominal massage and mice are sacrificed. Bladder and kidneys were harvested, and bacterial burden per milliliter urine or per gram tissue was determined. The amounts and concentrations of adjuvant used for each vaccine were determined on the basis of predetermined values as noted in product specification sheets and in published studies (75–78). A total of five adjuvants were tested for their efficacy within the vaccine formulations: aluminum hydroxide gel (alum) (Alhydrogel adjuvant 2%, InvivoGen), polyinosinic-polycytidylic (C) [poly (I·C) high molecular weight (HMW)] (InvivoGen), monophosphoryl lipid A from S. enterica serovar Minnesota R595 (MPLA-SM) (InvivoGen), unmethylated CpG synthetic oligodeoxynucleotide (ODN) 2395, type C (CpG) (InvivoGen), and double mutant heat-labile toxin 1LT(R192G/L211A) (dmLT) (provided by John D. Clements and Elizabeth Norton, Tulane University School of Medicine) (79). See Table S2 in the supplemental material for the dose of adjuvant administered for each immunization route. For each vaccine trial, a control vaccine formulation was prepared containing adjuvant alone and phosphate-buffered saline (PBS) (8 g/liter NaCl, 0.2 g/liter KCl, 1.44 g/liter Na_2_HPO_4_, 0.24 g/liter KH_2_PO_4_, pH 7.4) with 1 mM EDTA. Prepared antigen formulations were administered to 7-to-8-week-old female CBA/J mice IN (20 μl/mouse, 10 μl/nare), IM (50 μl/mouse), or SQ (70 μl/mouse).

10.1128/mBio.00555-20.6 TABLE S2 Doses for each adjuvant by route of administration. Download Table S2, DOCX file, 0.02 MB. Copyright © 2020 Forsyth et al. 2020 Forsyth et al. This content is distributed under the terms of the Creative Commons Attribution 4.0 International license.

### Murine model of ascending UTI.

Female CBA/J mice were transurethrally challenged as previously described (80). Briefly, bacterial pellets were harvested with centrifugation (3,000 × *g*, 30 min, 4°C) and resuspended in PBS to a final dose of 2 × 10^9^ CFU/ml. The inocula used for the experimental trials are compared in Fig. S1 in the supplemental material. Each mouse was anesthetized and transurethrally infected with 50 μl of bacterial suspension using a Harvard apparatus with a flow rate of 100 μl/min. At 48 h postinoculation, blood and urine were collected, mice were euthanized, and bladder and kidneys were harvested. Urine and organ homogenates were diluted, plated on LB agar using an Autoplate 4000 spiral plater (Spiral Biotech), and enumerated using a QCount automated plate counter (Spiral Biotech) to determine the CFU counts per milliliter urine or CFU counts per gram tissue.

### Antibody quantification by ELISA.

Quantification of antigen-specific antibody concentrations via indirect enzyme-linked immunosorbent assay (ELISA) was performed as previously described (20). Briefly, 5 μg/ml purified protein diluted in bicarbonate/carbonate buffer (3.03 g/liter Na_2_CO_3_, 6.0 g/liter NaHCO_3_) was coated in each well and incubated at 4°C overnight. Plates were washed with PBST (PBS containing 0.05% Tween 20) using an ELx405 microplate washer (Bio-Teck Instruments, Inc.) and blocked with SuperBlock (Pierce). Following a second wash in PBST, 50 μl of sera diluted in SuperBlock or undiluted urine was added to wells and incubated for 1 to 2 h at room temperature. Plates were again washed with PBST and coated with 50 μl 1:10,000-diluted secondary antibody goat anti-mouse IgG (horseradish peroxidase [HRP] conjugated) (catalog no. 1030-05; SouthernBiotech) and incubated 1 h at room temperature. After a final wash in PBST, 50 μl 1-Step Ultra TMB (3,3′,5,5′-tetramethylbenzidine) (Thermo Scientific) was added to each well and incubated at room temperature until sufficient color had developed. To stop the reaction, 50 μl 2 M sulfuric acid was added to each well and the absorbance at 450 nm was read with a μQuant plate reader (Bio-Tek Instruments, Inc.). Antibody concentrations were determined by comparing absorbance values to known concentrations of mouse IgG (catalog no. 0107-01; SouthernBiotech) bound to the plate with goat anti-mouse Ig (catalog no. 1010-01; SouthernBiotech). Serum assays were performed in duplicate for each mouse.

### Statistical analysis.

All graphic images and statistics were generated with Prism version 7 (GraphPad Software, Inc.). Significant differences in colonization levels and in numbers of mice without detectable CFU were determined by a two-tailed Mann-Whitney test and Fisher’s exact test, respectively. Correlations between antibody concentrations and bacterial burden were determined by Pearson’s correlation coefficient.
