# Supplementary figures and images for: Optimization of an Experimental Vaccine To Prevent Escherichia coli Urinary Tract Infection
Source: mBio. 2020 Apr 28;11(2):e00555-20. doi: 10.1128/mBio.00555-20 (PMC7188996; doi:10.1128/mBio.00555-20)

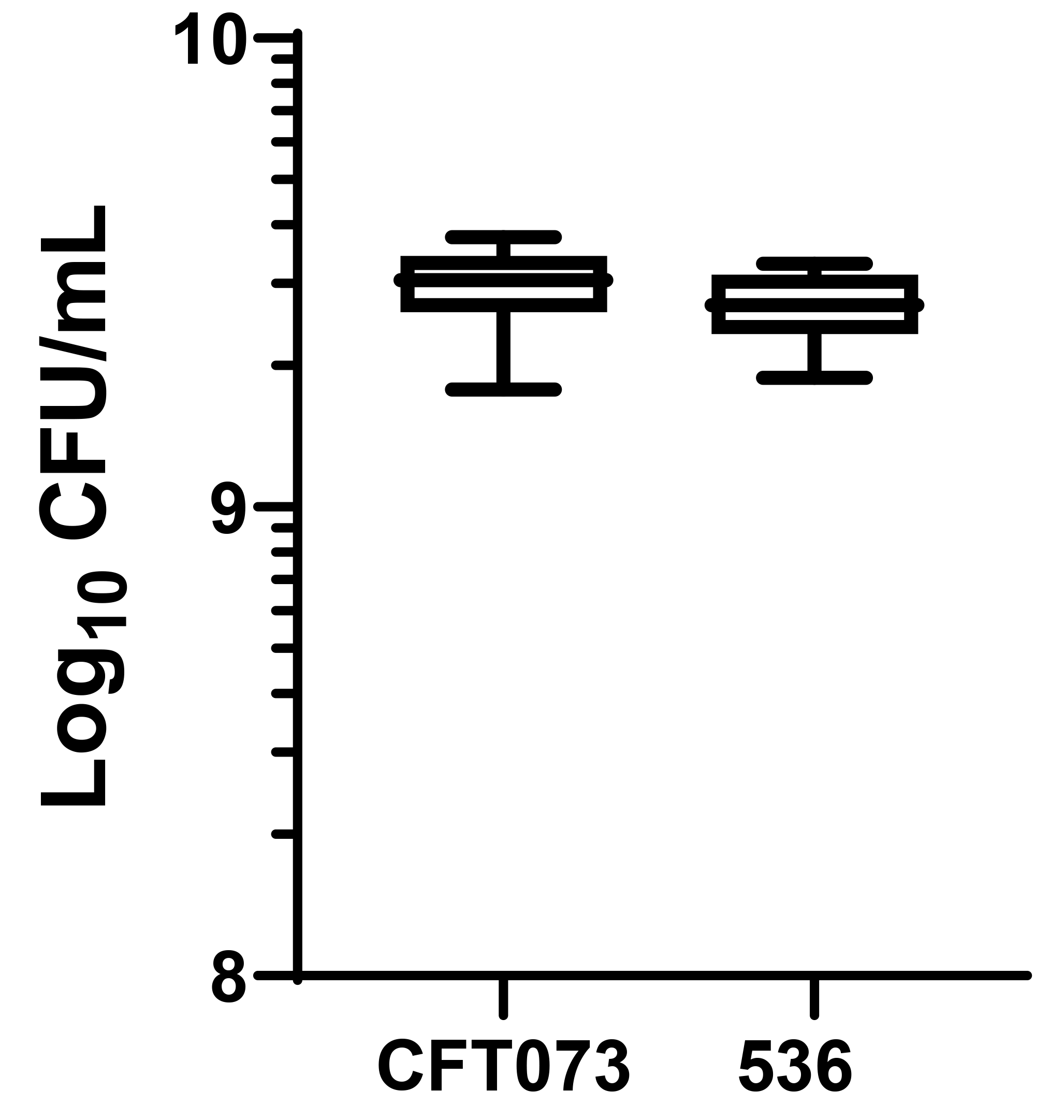

Supplement: FIG S1 [file mBio.00555-20-sf001.tif]

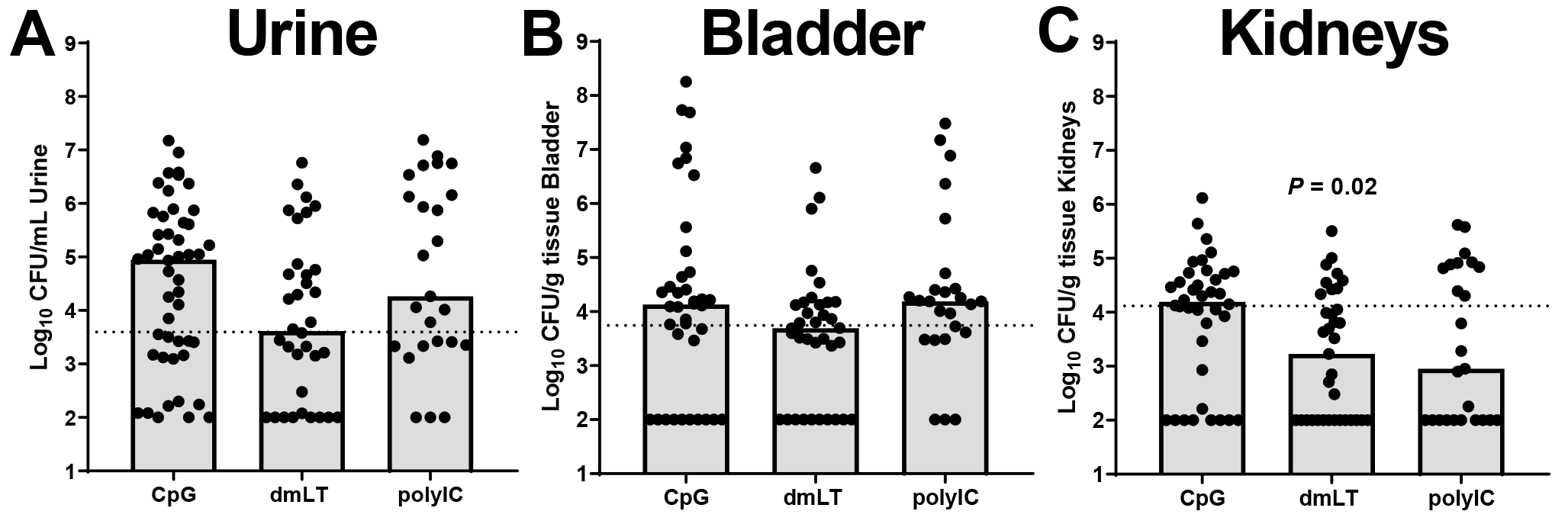

Supplement: FIG S2 [file mBio.00555-20-sf002.tif]

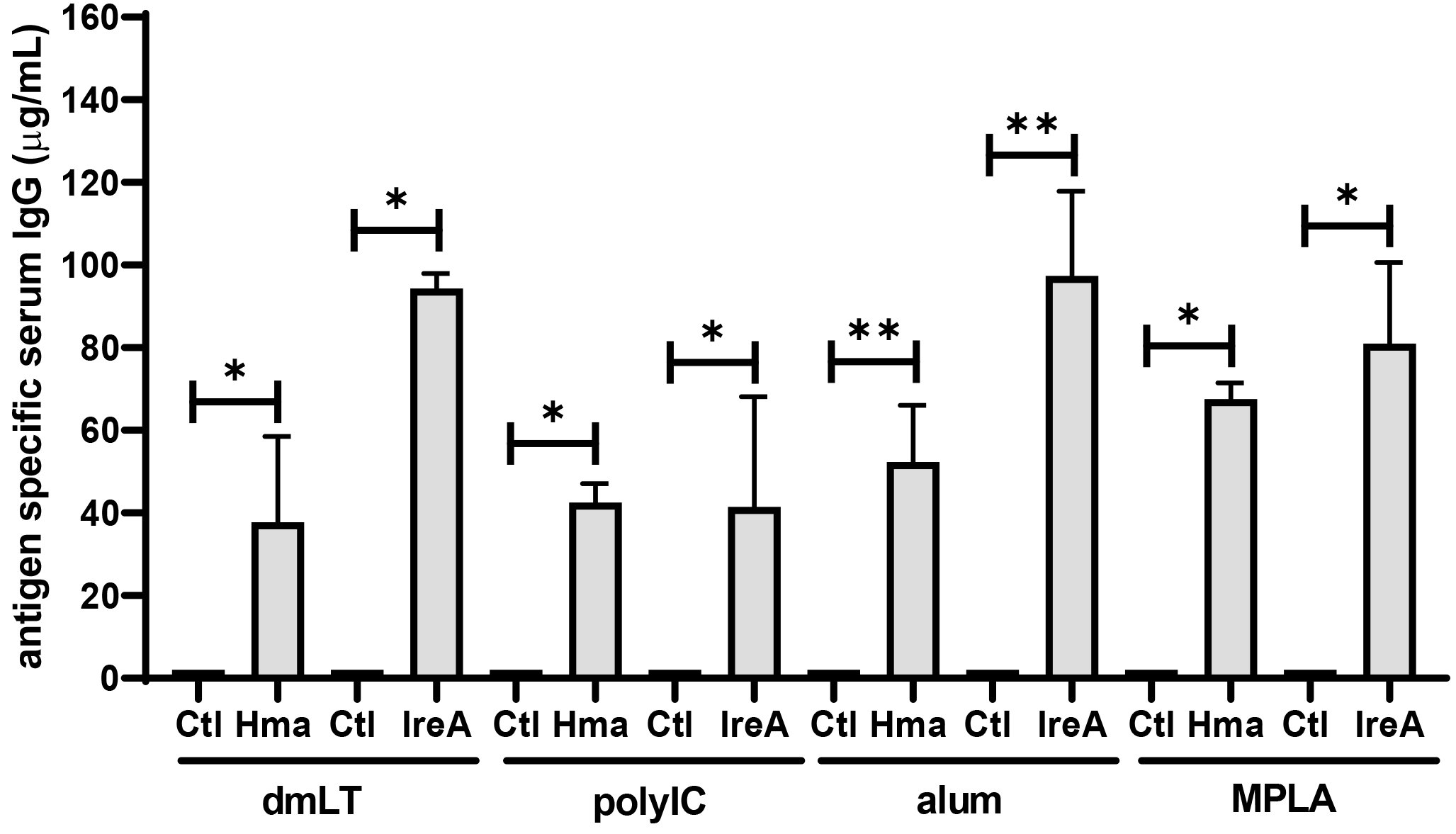

Supplement: FIG S3 [file mBio.00555-20-sf003.tif]

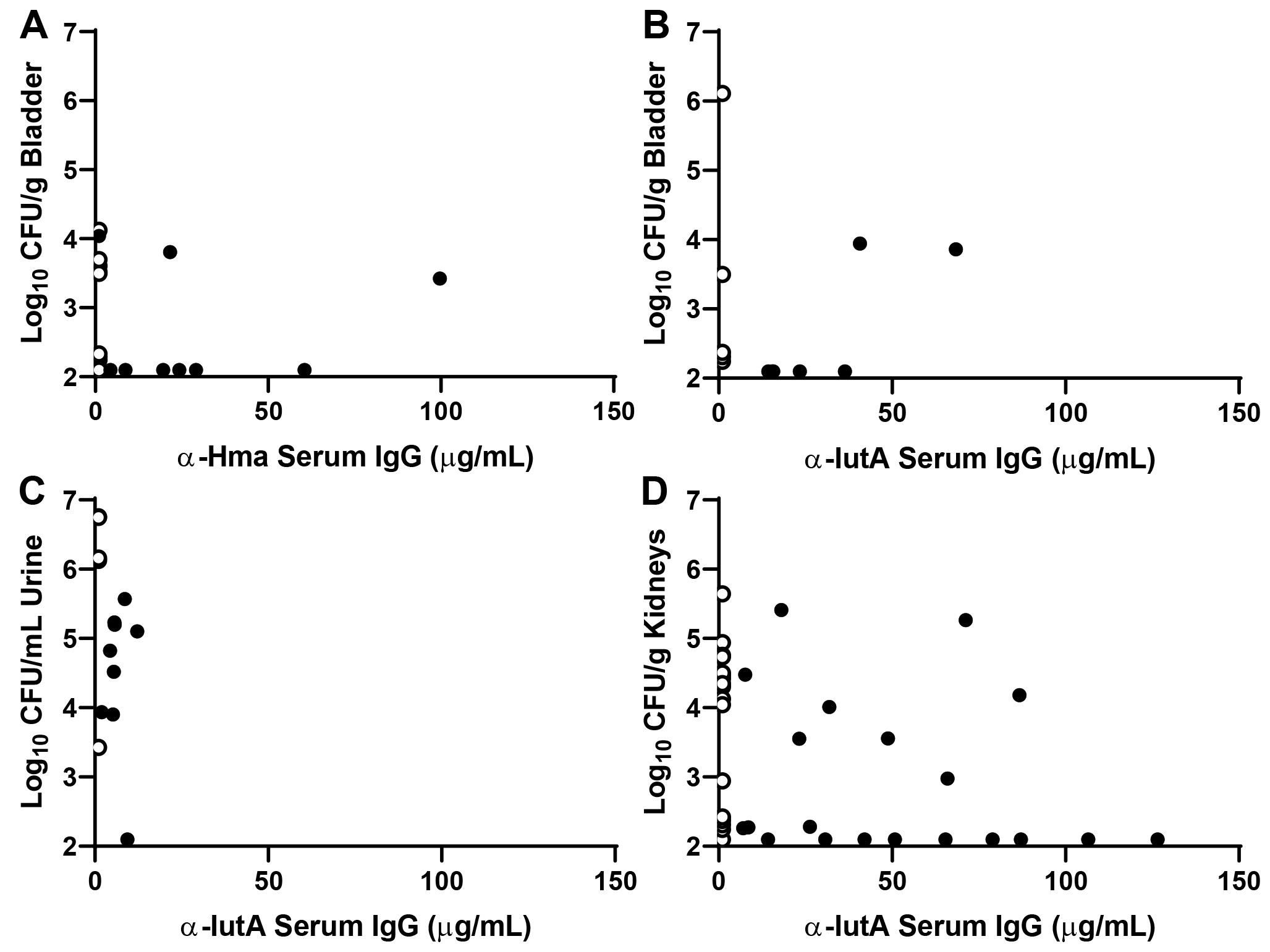

Supplement: FIG S4 [file mBio.00555-20-sf004.tif]
