# Supplementary material for: Optimization of an Experimental Vaccine To Prevent Escherichia coli Urinary Tract Infection
Source: mBio. 2020 Apr 28;11(2):e00555-20. doi: 10.1128/mBio.00555-20 (PMC7188996; doi:10.1128/mBio.00555-20)
Supplement: TABLE S1 [file mBio.00555-20-st001.docx]

**
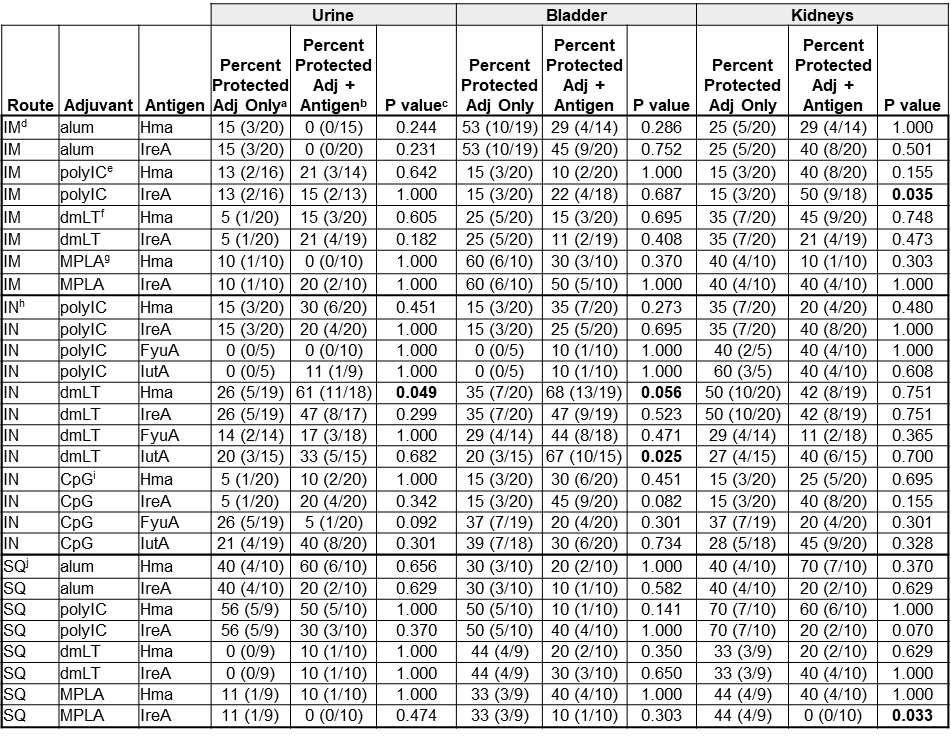
Table S1. Percent of immunized mice without detectable CFU following transurethral challenge**

^a^Percent of total number of mice without detectable CFU when immunized with the adjuvant alone. Number of mice without detectable CFU out of the total number of mice per group shown in parentheses.

^b^Percent of total number of mice without detectable CFU when immunized with the adjuvant formulated with antigen. Number of mice without detectable CFU out of the total number of mice per group shown in parentheses.

^c^*P* value as determined by Fisher's exact test. Significant differences are shown in bold.

^d^Intramuscular ^e^Polyinosinic:polycytodylic acid ^f^Detoxified *E. coli* enterotoxin ^g^Monophosphoryl lipid A ^h^Intranasal

^i^Unmethylated CpG synthetic oligodeoxynucleotides ^j^Subcutaneous
