# Supplementary material for: Optimization of an Experimental Vaccine To Prevent Escherichia coli Urinary Tract Infection
Source: mBio. 2020 Apr 28;11(2):e00555-20. doi: 10.1128/mBio.00555-20 (PMC7188996; doi:10.1128/mBio.00555-20)
Supplement: TABLE S2 [file mBio.00555-20-st002.docx]

**Table S2. Dose for each adjuvant by route of administration**


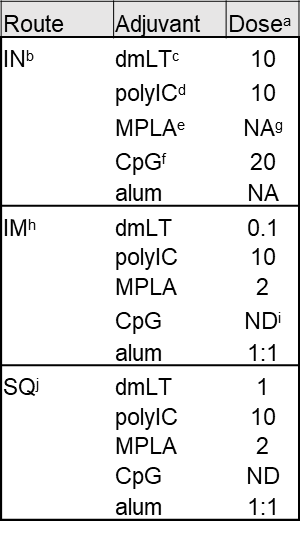


^a^Amount of adjuvant (µg/mouse or v:v) administered via the indicated route.

^b^Intranasal

^c^Detoxified *E. coli* enterotoxin

^d^Polyinosinic:polycytodylic acid

^e^Monophosphoryl lipid A

^f^Unmethylated CpG synthetic oligodeoxynucleotides

^g^Adjuvant is not approved for use in mice via the indicated route.

^h^Intramuscular

^I^Not tested

^j^Subcutaneous
